# The Features of Mobile-Based Software in Self-Management of Patients with Asthma: A Review Article

**Published:** 2020-01

**Authors:** Hassan Emami, Farkhondeh Asadi, Ali Garavand

**Affiliations:** Department of Health Information Management and Technology, School of Allied Medical Sciences, Shahid Beheshti University of Medical Sciences, Tehran, Iran

**Keywords:** Asthma, Self-management, Software, M-health

## Abstract

**Background:**

The use of mobile-based software for the self-management of patients with asthma improves the quality of life, reduces healthcare costs, provides effective health care interventions in asthma, and supports the patients in self-management. The current study was performed to identify the features of mobile-based self-management software for patients with asthma (MSSPA).

**Materials and Methods:**

The present review study was performed in 2018. Four databases including PubMed, Scopus, Emerald, and Google Scholar were screened by the combination of selected keywords. Data were collected using a data extraction form. Data were analyzed using the content analysis method. Results were abstracted and reported based on the study objectives.

**Results:**

Of the 297 articles retrieved during the first round of search, 24 were selected; 15 of which were the original articles (62.5%). As the most important applications of MSSPA, it could be used as a tool to support patients in self-management, provide them with educational information, and self-observation. Also, 75% of the studies (n=18) emphasized the effectiveness of MSSPA. Identification of the required field of the software was the most important requirement in using MSSPA. Nevertheless, some of the studies reported the low quality and compatibility of some designed apps compared with those of the available information systems.

**Conclusion:**

Identification of MSSPA features and considering them in new versions can promote the quality of MSSPA. However, according to the results of the study, in addition to identifying the software features, more attention should be paid to the users’ needs in software design.

## INTRODUCTION

Asthma is one of the most common chronic respiratory diseases associated with inflammation and repeated cough attacks, shortness of breath, and chest wheezing. Also, the intensity and frequency of its symptoms vary across patients (1–3). Asthma is commonly attributed to genetic predisposition, allergic inflammation, secondhand smoking, obesity, malnutrition, etc. (4–9). Asthma is characterized by reversible airway obstruction resolved spontaneously or by therapeutic measures. The stenosis of airway is usually reversible, but therapeutic measures are required in some patients with chronic asthma (6–10). The goals of asthma treatment include prevention of the symptoms, maintaining normal lung function, supporting the patient to recover normal performance, prevention of the disease relapse, providing the best medicine treatment with the fewest side effects, and increasing the satisfaction of the patient and his family (11). Different methods are used to manage asthma, including drug therapy, hospital stay, monitoring, and self-management by patients and their families to improve patients’ health status (12–14). Although the employment of appropriate strategies for asthma management can lead to reduced mortality rate, some new policies should be adopted (15). Asthma self-management is a systematic method of patient education to control and manage the disease, which in many cases enhances the patient’s abilities and increases his\her experiences in self-management (16–19). Since self-management of asthma, as a chronic disease, needs daily management by the patient (20), poor implementation of the control programs may lead to increased mortality rate; however, some studies emphasized the weaknesses of such programs (21).In recent years, information technology (IT) is increasingly used in asthma self-management as a common supportive tool (22). The most important type of such technologies is used in mobile phones (23). Mobile technology intervention (MTI) is a common tool in the management of chronic diseases, and is usually used in drug therapy monitoring, recording of symptoms, and activation of disease management action plans (24). Today, the mobile phone is an integral part of people’s lives considering its ease of use, appropriateness, availability, and feasibility of releasing technologies and developing capacities and applications (25). The results of studies showed that the use of software and mobile-based applications in self-management of patients with asthma improved patients’ quality of life, reduced health care costs (26), supported them in self-management (27, 28), etc. Considering the large number of studies performed in this field (use of software and mobile-based applications), the importance of review and classification of the features of software, programs, and applications in asthma self-management, and the lack of a comprehensive review in this field, the present study aimed at identifying the features of mobile-based self-management software for patients with asthma.

## MATERIALS AND METHODS

### Study Selection:

The current review study was performed in 2018 to identify the features of MSSPA. The searches were conducted through PubMed, Scopus, Emerald, and Google Scholar databases using the combination of keywords shown in Table 1. All the review and original articles available with full texts and relevant topics were searched and reviewed in the current study. Also, all articles published in non-English languages, with unavailable full texts, irrelevant topics, and low quality were excluded from the study. All the study steps were conducted in accordance with PRISMA statement. The searches were performed by two researchers independently in order to prevent possible bias. In case of bias, contradictions were resolved by consulting with a third party.

**Table 1.** Search strategy of study

Search Databases: PubMed, Google Scholar, Scopus, Emerald	
Date: 1 Jan 2013-10 April 2018	
Search strategy: #1 AND #2 AND #3	
#1	(“Mobile health” OR “M Health” OR “mobile” OR “tablet” OR “portable devices” OR “Application” OR “mobile software*” OR “App*”)
#2	(“Asthma”)
#3	(“Self-management”)

### Inclusion and Exclusion Criteria:

In the current study, articles published within the past five years (2013–Feb 2018) were retrieved. All the articles on mobile-based software in asthma self-management and MSSPA applications were selected. Papers published on the technical phase of software or app design were excluded. Studies on the use of mobile-based software for purposes other than asthma management were not included. Other article types-e g, letter to editors, conferential papers, and short articles (short communication), were excluded.

### Data Extraction:

Data were collected using a data extraction form with four main parts including general information (author’s name, tittle, article type, publication year, study population, year of conduction, and place of study), methodological data (sample size, study type, and statistical analysis tests), the most important features (applications, requirements, effects, and limitations of MSSPA), and other important results of the study. Data were analyzed using the content analysis method. Results were reported based on the study objectives.

## RESULTS

**Figure 1.**
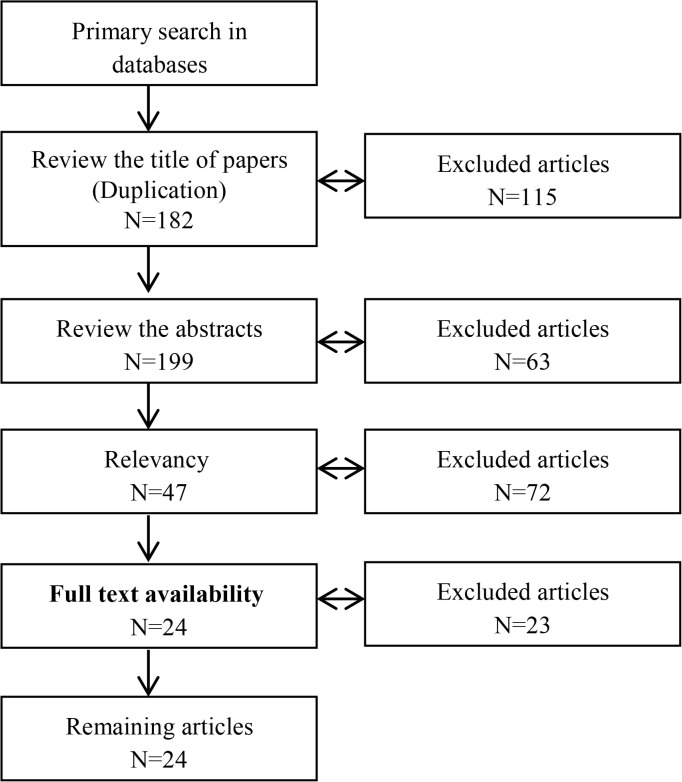
The process of articles selection based on PRISMA

Of the 297 articles retrieved during the first stage of the search, considering the inclusion and exclusion criteria, finally 24 articles were selected in the current study (Figure 1). The characteristics of all the 24 articles selected in the study are shown in Table 2.

**Table 2.** All selected articles in the study

row	First author name	Study title	population	Article type	Main results
1	Marcano, et.al. (21)	Smartphone and tablet self-management apps for asthma.		Review article	Improvement of lungs of affected person, there is no difference in self-management providing
2	Miller et.al. (24)	Mobile Technology Interventions for Asthma Self-Management: Systematic Review and Meta-Analysis.	11 articles reviewed	Meta-Analysis	MTI (Mobile Technology Intervention) has many effects on adherence to drug use and clinical outcomes, its noted to do more studies in this field of study
3	Cook et.al. (27)	Improvement in Asthma Control Using a Minimally Burdensome and Proactive Smartphone Application	60 elderly people	Four month cohort	Improvement in asthma control test score, intention to use of mobile technologies in all age groups, improvement of diseases control in uncontrolled cases
4	Farzandipour et.al. (29)	Patient Self-Management of Asthma Using Mobile Health Applications: A Systematic Review of the Functionalities and Effects	10 selected articles	Systematic review	Control of disease, improvement in patients quality of life
5	Hosseini et.al. (30)	Feasibility of a Secure Wireless Sensing Smart watch Application for the Self-Management of Pediatric Asthma	Children and elderly	developmental	Use in smart monitoring, the evaluation of risk level of asthma attack, the developer should note the HIPAA security rules
6	Tinschert et.al. (31)	The Potential of Mobile Apps for Improving Asthma Self-Management: A Review of Publicly Available and Well-Adopted Asthma Apps.	The evaluation of 38 apps	Systematic review in apps	Low quality of some of apps, more of them used in follow-up and providing information, ability to behavior change, most type of apps were android operating system
7	Simpson et.al. (32)	Perspectives of patients and healthcare professionals on mHealth for asthma self-management.	23 interviews with patients and physicians	qualitative	These software is useful for patients to monitoring and data gathering, and useful for specialists as a reminder for patients and Drug recommendation
8	Sage et.al. (33)	A Self-Regulation Theory-Based Asthma Management Mobile App for Adolescents: A Usability Assessment.	Teenagers aged 11–18 with asthma	qualitative	Charts and Alerts, Linear Chart of Treatment Process, there is high intention to use among patients
9	Hui et.al. (34)	The use of mobile applications to support self-management for people with asthma: a systematic review of controlled studies to identify features associated with clinical effectiveness and adherence.	12 selected RCT articles	Systematic review	Evaluation, monitoring, electronic notepad, Operational plans, drug reminder, Facilitate professional support, increase the patients awareness of asthma and asthma control, and decision support for physicians
10	Carpenter et.al. (35)	Exploring the theoretical pathways through which asthma app features can promote adolescent self-management	20 teenager aged 16–20 with asthma	Qualitative	The effectiveness in self-observation, self-assessment, self-efficacy, goal setting, drug reminder, Monitoring drug use, providing self-assessment tests to control of asthma
11	Panzera et.al. (36)	Adolescent asthma self-management: patient and parent-caregiver perspectives on using social media to improve care.	18 patients and 18 care giver	Qualitative	Following the condition, management in drug use, reducing absenteeism in school
12	Licskai et.al. (37)	Development and pilot testing of a mobile health solution for asthma self-management: asthma action plan smartphone application pilot study	22 elderly people	Observational	Use in current situation control, Notification of air quality in the special days, follow-up the self-management advices and reduce in available risks.
13	Hollenbach et.al. (38)	Understanding clinicians’ attitudes toward a mobile health strategy to childhood asthma management: A qualitative study.	41 elderly people	qualitative	Use in booking in Clinics is one of the physician’s demands of these apps. Lung specialists want to evaluate the performance lung through spirometry and examine the lung function at various visits and with their data. But pediatrics do not welcome it
14	Zairina et.al. (39)	Telehealth to improve asthma control in pregnancy: A randomized controlled trial	72 pregnant women who were asthmatic	RCT	Improvement in quality of life, better control of lung performance in first 6 months, daily program, don’t support of oral intake of corticosteroids in perinatal period
15	Househ et.al. (40)	A cross-sectional content analysis of Android applications for asthma	15 apps about asthma	Systematic review in apps	Positive effect on patients self-management, Providing conditions for better education, the future studies should be done in monitoring of patients by these apps
16	Peters et.al. (41)	Young People’s Preferences for an Asthma Self-Management App Highlight Psychological Needs: A Participatory Study.	Young people aged 15–24 years with asthma	qualitative	Note to people mental condition in creation of apps is a key factor to successful use of them
17	Stukus et.al. (42)	Real-world evaluation of a mobile health application in children with asthma	98 patients between 6 month to 21 years old	RCT	Has a significant effect on reducing the frequency of emergency referral, reduce the length of stay of patients
18	Roberts et.al. (43)	Adolescent, caregiver, and friend preferences for integrating social support and communication features into an asthma self-management app	20 patients (12–16 years old), 20 health care provider and 3 of patients friends	Qualitative	It is a supportive tool to patients and physicians, the teens trust it and involving teenagers’ friends in their self-management
19	Nguyen et.al. (44)	Integrated Self-Management System for Improved Treatment of Asthma		Developmental	Support in self-assessment in home, office or leisure time
20	Huckvale et.al. (45)	Apps for asthma self-management: a systematic assessment of content and tools	103 selected apps	Systematic review in apps	Providing information about patient conditions, it should provide comparative information, Careful in their use
21	McLean et.al. (46)	Interactive digital interventions to promote self-management in adults with asthma: systematic review and meta-analysis.	3 articles	Meta-analysis	Although positive effects have been reported, more studies are needed to get more accurate results
22	Perry et.al. (47)	Smartphone-based vs paper-based asthma action plans for adolescents.	44 elderly people in a 6 month period of time	RCT	Positive effect on self-management, suggest to further studies, it’s hard to use by elderly people and satisfying to continuing to its use is very low
23	Joseph et.al. (48)	Evaluation of a web-based asthma management intervention program for urban teenagers: Reaching the hard to reach	422 students from a particular minority	RCT	Control of asthma in Poor communities
24	Baptist et.al. (49)	Technology-Based Interventions for Asthma-Can They Help Decrease Health Disparities?	16 articles	Review article	It’s useful for patients with mild asthma and patients with low level income

The results showed that 18 articles (75%) focused on the effectiveness of mobile-based software in asthma self-management; six studies (25%) did not report differences between traditional methods and mobile-based software in asthma self-management. Eight studies (33%) were performed on young patients and five (21%) on the elderly; one article was about pregnant women with asthma.

**Figure 2.**
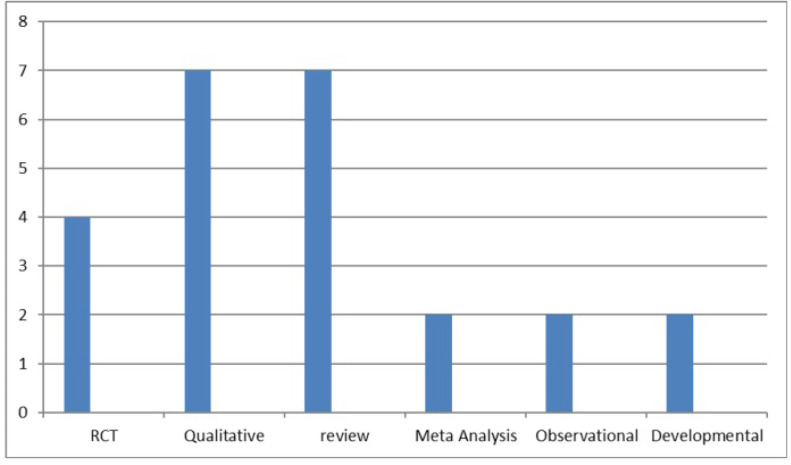
The frequency of types of selected articles

Findings of the study showed that seven studies (29.17%) were qualitative researches. Figure 2 shows the frequency of articles based on the type.

The results of the study also showed that in addition to patients and physicians, healthcare providers and patients’ families and friends were other individuals affected by such the software.

Regarding the study objectives, the features of MSSPA were categorized into four dimensions including applications, requirements, effects, and limitations.

**A) Applications**

The applications are shown in Table 3.

**Table 3.** The applications of MSSPA

	**Applications**
Control of asthma disease	Control of diseases (include the evaluation of risk level, change in behaviors, adherence to drug use and alert system) (24,27,29–36)
Providing useful information	Providing educational information to patients (29,34,37), The ability to information sharing (29,34,35,38), Data gathering and reporting (32,34,38)
patients’ monitoring	Patient condition monitoring (32,35)
appointment time and e-booking	e-booking (33,38)
self-assessment and self-observation of patients	Self-assessment and self-observation (35), Prescription (32)
Supporting decision making	Decision support (34), Assessment of lungs efficiency (38),

According to Table 3, the main application of MSSPA was in disease control.

**B) Requirements**

The requirements are mentioned in the second part of the study results. Identifying the MSSPA requirements is important. According to the goals of the Health Insurance Portability and Accountability Act (HIPAA) (31), conducting further studies to obtain more definitive results (39,40), paying more attention to patients with mental conditions (41), identifying the fields required to the software (33), as well as its interactivity and dynamicity (37), and training the elderly and other individuals in using mobile should be considered (34,37).

**C) Effects**

The study results also showed that reduced patients’ length of stay in a hospital was the most effective application of the software (30, 43), followed by improved communication between patients and physicians (43), enhanced quality of healthcare services (39), reduced asthma-related school absenteeism(36), lowering the risks (36), and reduced referring to healthcare centers(42).

**D) Limitations**

The results of the study showed that in some cases, the software had a low quality and compatibility, compared with those of the available information systems (31, 40).

## DISCUSSION

Today, various potentials of m-health are used to provide healthcare services to patients with chronic diseases (25), especially the ones performing self-management. Many studies highlight the effectiveness and usefulness of m-health. It is used by different age groups of patients performing self-management in different parts of the world; various studies introduce the features of m-health. The most important features of MSSPA can be categorized into four dimensions including applications, requirements, effects, and limitations discussed as follows:

Applications

- **1. Controlling of asthma**
  Based on the results of the study, MSSPA could be applied to control the disease, particularly in uncontrolled cases. Cook et al. in a study concluded that mobile-based applications can be used as an effective tool to control uncontrolled asthma (27). Another study showed that physicians are more inclined to use m-health tools as an alert system for patients under critical conditions (32).
  It seems that the prescription of mobile-based software, with the required standards and permissions, to patients with uncontrolled asthma can be a good measure for self-management of the disease.
- **2. Providing useful information**
  The results of the study showed that 16.66% of the selected articles recommended the use of MSSPA by patients in order to obtain useful information. Therefore, the instructions and guides for asthma self-management provided in the software can be used by patients, and then they independently can take place in the self-management program. Hai in a study noted the employment of mobile-based applications in patient education as one of the seven main features of MSSPA (34). Therefore, it is suggested to embed educational information about asthma in mobile-based software used by patients for self-management purposes.
- **3. Monitoring of patients**
  Patients with asthma have chronic and sensitive conditions, which require more caring and monitoring, which highlights the use of MSSPA for this purpose. Simpson et al. concluded in their study that the use of such apps can be useful in monitoring the patients with asthma (32). Also, Carpenter et al. reported that the monitoring the patients is one of the main goals in using the self-management applications (35).
- **4. Scheduling appointments and e-booking**
  In some studies, the feasibility of scheduling doctor’s appointments and e-booking is considered as the main application of MSSPA, which is a benefit to both patients and physicians (38). Embedding such an option in mobile-based applications helps the patients schedule their appointments with no anxiety.
- **5. Self-assessment and self-observation of patients**
  According to the study findings, some researchers emphasized self-assessment and self-observation of patients (35, 43). Self-assessment and self-observation involve patients in treatment trends and sensitize them to their situation via assessing health conditions; as a result, the MSSPA can improve self-management in patients. Using such apps, physicians can better monitor the patient’s health status, prescribe drugs, and give advice (32).
- **6. Supporting decision making**
  Supporting decision making is one of the applications of this software for physicians (34). This capability can encourage physicians to employ MSSPA. Despite its difficulties, producing software with such options seems essential.
- **7. Requirements**
  There are many requirements for MSSPA, and more attention should be paid to them in software development. The results of the study showed the high importance of the security and privacy of such applications. Tinschert et al. indicated that paying more attention to HIPAA goals is one of the main requirements of MSSPA design (31). Another important requirement includes considering the patient’s health status. The study by Peters et al. showed that paying more attention to patients’ mental health is of great importance in MSSPA design (41). Zairina et al. in a study evaluated the utilization of tele-health tools in the control of pregnant women with asthma (39).

Other results showed that such apps should possess the feasibility of sharing the data of adolescents with asthma with their parents as it can support them both financially and technically, and in turn improve patient’s self-management (35). Therefore, this option seems essential for this group of patients.

### Effects

The current study results showed that 75% of the selected articles emphasized effectiveness of mobile-based software in the improvement of self-management of patients with asthma from the viewpoint of both physicians and patients.

Also, it was shown that the utilization of MSSPA can reduce the length of hospital stay, improve communications between patients and physicians, enhance the quality of care and patient’s quality of life, and reduce asthma-related school absenteeism, risk of asthma, and rate of referring to hospitals. (30,35,36,39,42).

Many of the articles reviewed in the current study emphasized the positive effects of software use on self-management of patients with asthma. However, some studies noted disadvantages of the software. For example, Huckvale et al. showed that because of safety issues associated with software application, the developers should pay more attention to its security (45). Also, Mclean et al. suggested that more studies should be performed in this field to achieve accurate and valid results (46). Miller et al. concluded that MTI has a positive effect on adherence to drug use and treatment regimen, although there was no significant difference between MTI and traditional methods; further studies in this field is recommended (24).

### Limitations

The results of the study showed that in some articles the software has a low quality and compatibility compared with the available information systems (31, 40). Neglecting various professions in the development and use of software, the lack of real users’ need assessment, incorrect evaluations, and purely a commercial view in software production can be the main limitations of the produced software.

## CONCLUSION

It should be noted that there are big differences between the patients and physicians point of views in using MSSPA. It is necessary to integrate the requirements and comments of the users, and apply them to the design of a standard program in order to help patients, physicians, healthcare providers, and even patients’ families and friends. The specialists and health care providers should not be forgotten the self-management of them at the first level, and following that, they can programed to use of mobile-based software programs, because some of mobile-based software programs don’t have positive effects in self-management of patients with asthma. Based on the obtained results, further studies in this field are required in order to achieve more conclusive results about the effects of such software on patients with asthma. Also, the requirements of the users including physicians, patients, nurses, and healthcare providers should be considered.
